# Management of Giant Omphalocele Leading to Early Fascial Closure

**DOI:** 10.7759/cureus.5932

**Published:** 2019-10-17

**Authors:** Mustafa Erman Dörterler

**Affiliations:** 1 Pediatric Surgery, Harran University Faculty of Medicine, Şanlıurfa, TUR

**Keywords:** omphalocele, povidone, bandage, congenital malformations, non-operative management, antibiotic powder

## Abstract

Objective

The aim of the study was to present the clinical outcomes of patients diagnosed with giant omphalocele, treated with early primary closure combined with bandaging and povidone-iodine staining and powder spray antibiotics.

Materials and methods

The study included a total of 22 infants with omphalocele. The omphalocele sacs of the patients were cleaned daily with topical povidone-iodine. A powdered spray antibiotic combination was then applied topically twice a day and the sac was wrapped in a sterile elastic bandage. Following the observation of eschar formation and epithelization, the patients were operated on in the early period and the sac was removed by placing a graft.

Results

Evaluation was made of a total of 14 female and eight male patients with mean duration of conservative monitoring of 11 days and mean total hospital stay of 35. The mean giant omphalocele (GO) defect size of the patients followed-up was 10 cm. Since seven of the patients in the present study died in the 1^st^ week, conservative and elastic bandages were applied for the GO treatment of 15 patients for 9-14 days. After the conservative follow-up, it was determined that the patients who were applied with a graft at an average of 11 days were hospitalized for an average of 24 days postoperatively.

Conclusion

In conclusion, it is possible to reduce the length of hospital stay with primary closure in the early period by providing faster epithelialization with the combination of povidone-iodine and antibiotic powder together with elastic bandage application in infants with GO.

## Introduction

Omphalocele is one of the most common congenital abdominal wall defects seen in approximately one in 4000-7000 live births [1]. It is associated with an under-developed abdominal cavity and a high visceral-abdominal disproportion that prevents safe primary closure [2]. This is the midline defect of the abdomen where the abdominal organs and especially the bowel segments are herniated with a sac. Prenatal screening has enabled early detection of omphalocele and related anomalies and increased the frequency of elective termination application [3]. However, infants born with giant omphalocele (GO) still represent an important challenge in respect of the clinical approach in pediatric surgery practice.

The purpose of GO treatment is the closure of the abdominal wall defect after reducing the abdominal content and stabilization with supportive care. The GO treatment methodology is divided into two basic categories. The first and most commonly used method in recent years is non-operative delayed closure, which involves the maintenance of the sac with topical medications and regular dressings, providing epithelialization and closure of the ventral hernia with delayed surgery. The second method, however, is the removal of the graft and primary closure after ensuring epithelization with the graft in the early period [4-6].

Patients with GO have a small abdominal cavity and early closure of the abdominal wall can lead to a sudden increase in intra-abdominal pressure and respiratory failure due to reduced lung capacity. This is particularly alarming in patients with GO because these patients also tend to have pulmonary hypoplasia which may cause respiratory failure [7,8]. However, the abdominal-visceral disproportion in newborns, the large diameter of the abdominal wall defect, presence of large liver tissue in the sac and other organ anomalies coexisting in infants make early surgical treatment impossible [9,10].

The current main treatment strategy is the follow-up of the sac with a topical antimicrobial or escharotic agent, as well as a “stain and wait” approach with graft stepped or without graft closure following the development of the appropriate epithelial surface of the sac [11]. Non-surgical treatment of omphalocele with primary epithelialization is considered to be a good option, although it has a higher incidence of sepsis and needs correction of the ventral hernia later in life. In a recent review by Bauman et al., it was concluded that non-operative delayed treatment was associated with lower mortality compared to early stepped surgical correction and a shorter start to complete enteral feeding in the neonatal period [4].

There are many topical agents used in GO care. Silver sulfadiazine and povidone-iodine have unique advantages and disadvantages. The first is low cost and the facilitation of early granulation with good broad-spectrum antibiotic coverage. The disadvantage of silver sulfadiazine is that it has the potential to disrupt the granulation tissue. The disadvantage of povidone-iodine is that it leads to thyroid dysfunction. However, no clear evidence of the association of iodine exposure due to omphalocele with permanent hypothyroidism has been demonstrated [12].

While the non-operative delayed treatment has lower mortality rates and better clinical outcomes, it has some disadvantages such as a longer wait for an operation and difficulty in wound care during this period. The aim of this study was to present the clinical outcomes of patients diagnosed with GO with early primary closure combined with bandaging (3M™ Coban™) and povidone-iodine staining and powder spray antibiotics (Polymyxin B sulfate, Bacitracin zinc, and Neomycin).

## Materials and methods

Patient selection

A total of 22 infants were identified with GO on prenatal ultrasonography (US). The GO was defined as an omphalocele with a midline abdominal wall defect of >7 cm in size or containing a large segment of the liver within the sac.

Conservative monitoring

Patient characteristics and omphalocele measurements were recorded in the postnatal period after stabilization of the patients who were seen to have GO with US before delivery. The omphalocele content (liver, spleen, bowel segment, etc.) was recorded and necessary intervention was performed by evaluating the cases of rupture, infection, and sepsis.

**Figure 1:**
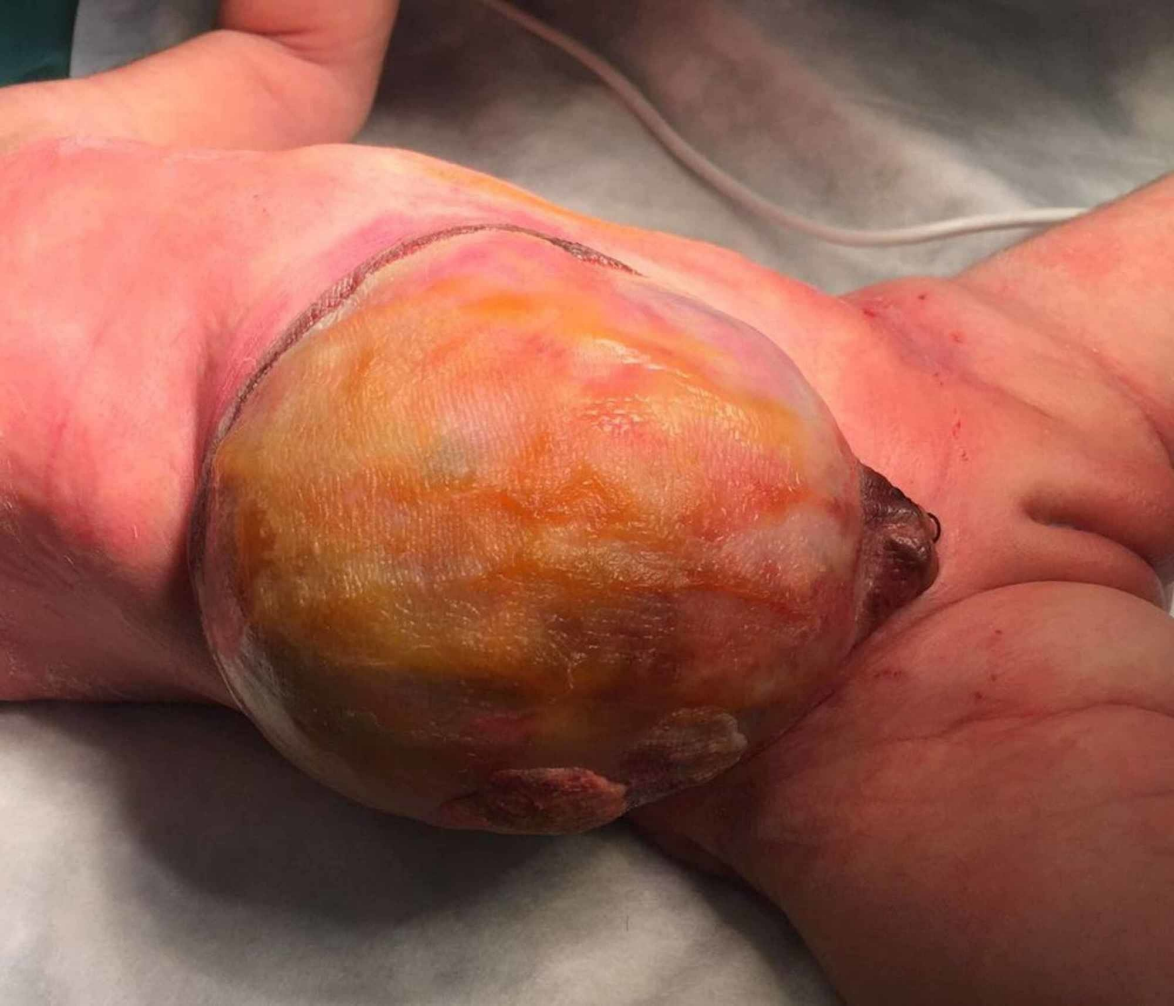
Staining of omphalocele with betadine and spraying the antibiotic mixture powder.

The omphalocele sacs of the patients were cleaned daily with topical povidone-iodine (5% solution) (Figure 1).

**Figure 2:**
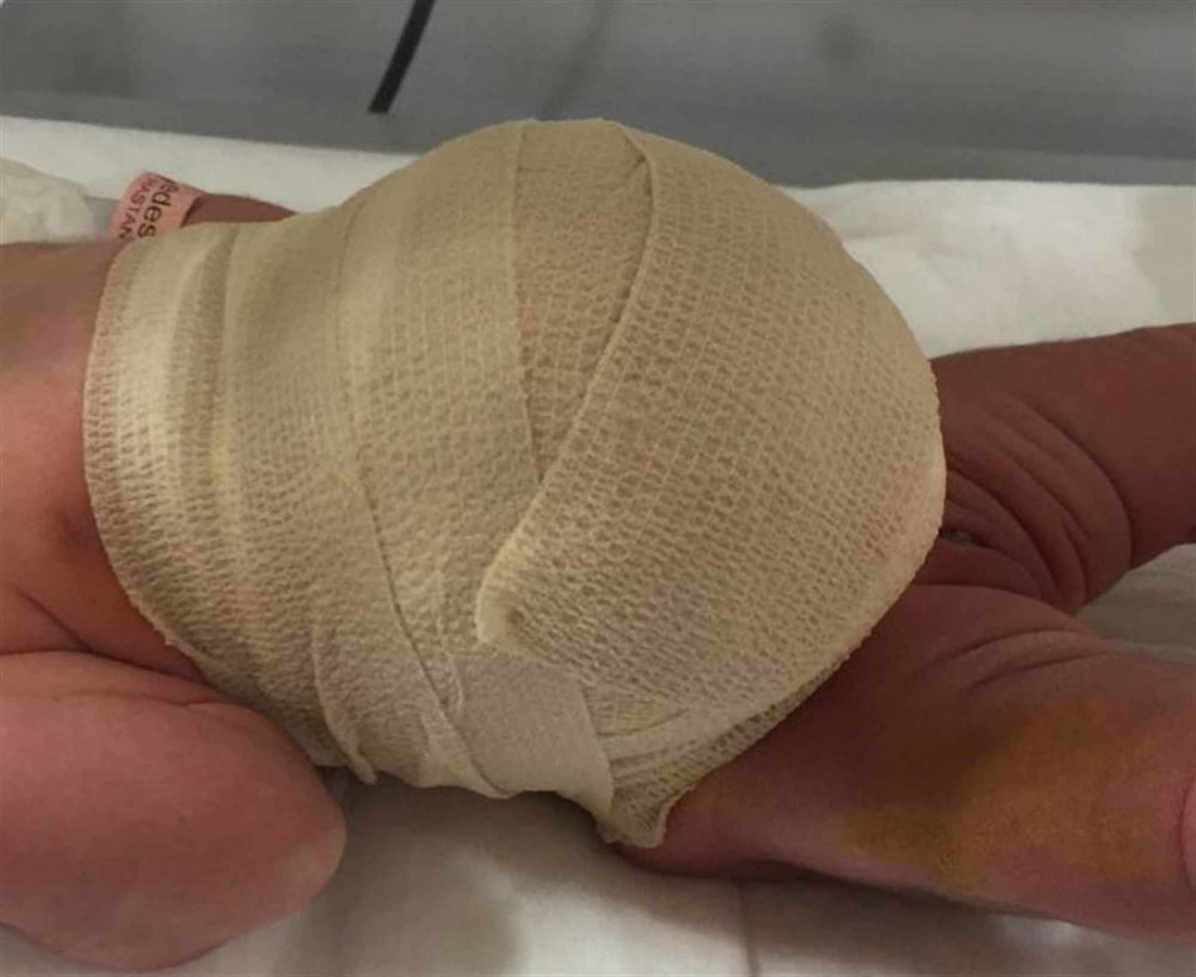
Elastic abdominal bandage application.

The powdered spray antibiotic combination (Polymyxin B sulfate, Bacitracin zinc, and Neomycin) was then applied topically twice a day and the sac was wrapped in a sterile elastic bandage (3M™ Coban™) (Figure 2) [13].

The elastic bandage was closed so that it covered the area between the infant’s xiphoid and pubis. In the cases with liver segment in the sac, an extra gauze patch was placed between the bandage and the fascia adjacent to the liver, and support was provided by pushing it from the midline. The topical application and bandage application were repeated twice a day. After each bandage application, the infants were monitored for two hours in terms of vomiting, tachycardia and respiratory distress, and were provided with breast milk and/or hyperalimentation fluid. The abdominal bandage was loosened or removed in cases with increased respiratory distress, restlessness or persistent vomiting. The omphalocele sacs were checked daily and re-epithelization and eschar formation were evaluated.

Graft application

**Figure 3:**
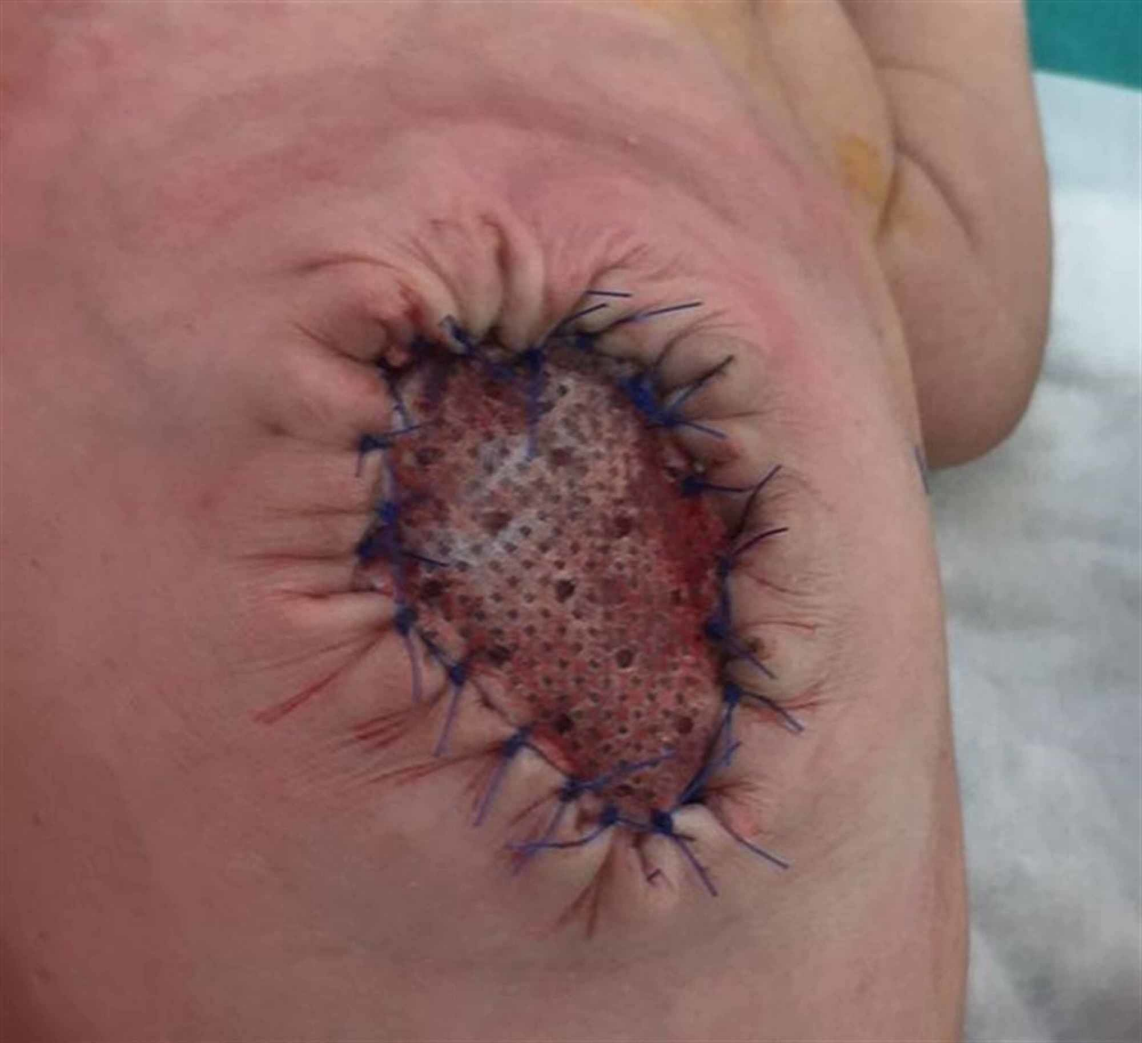
Closure of omphalocele with graft.

Following the observation of eschar formation and epithelization, the patients were operated on in the early period and the sac was removed by placing a graft (polyglactin 910 Woven Mesh & VICRYL®) (Figure 3).

Other procedures, if any, such as fundoplication, inguinal hernia repair or orchidopexy were performed at the same time as ventral hernia repair. The infants continued to be fed during this time and daily dressings were applied.

Primary closure and discharge

**Figure 4:**
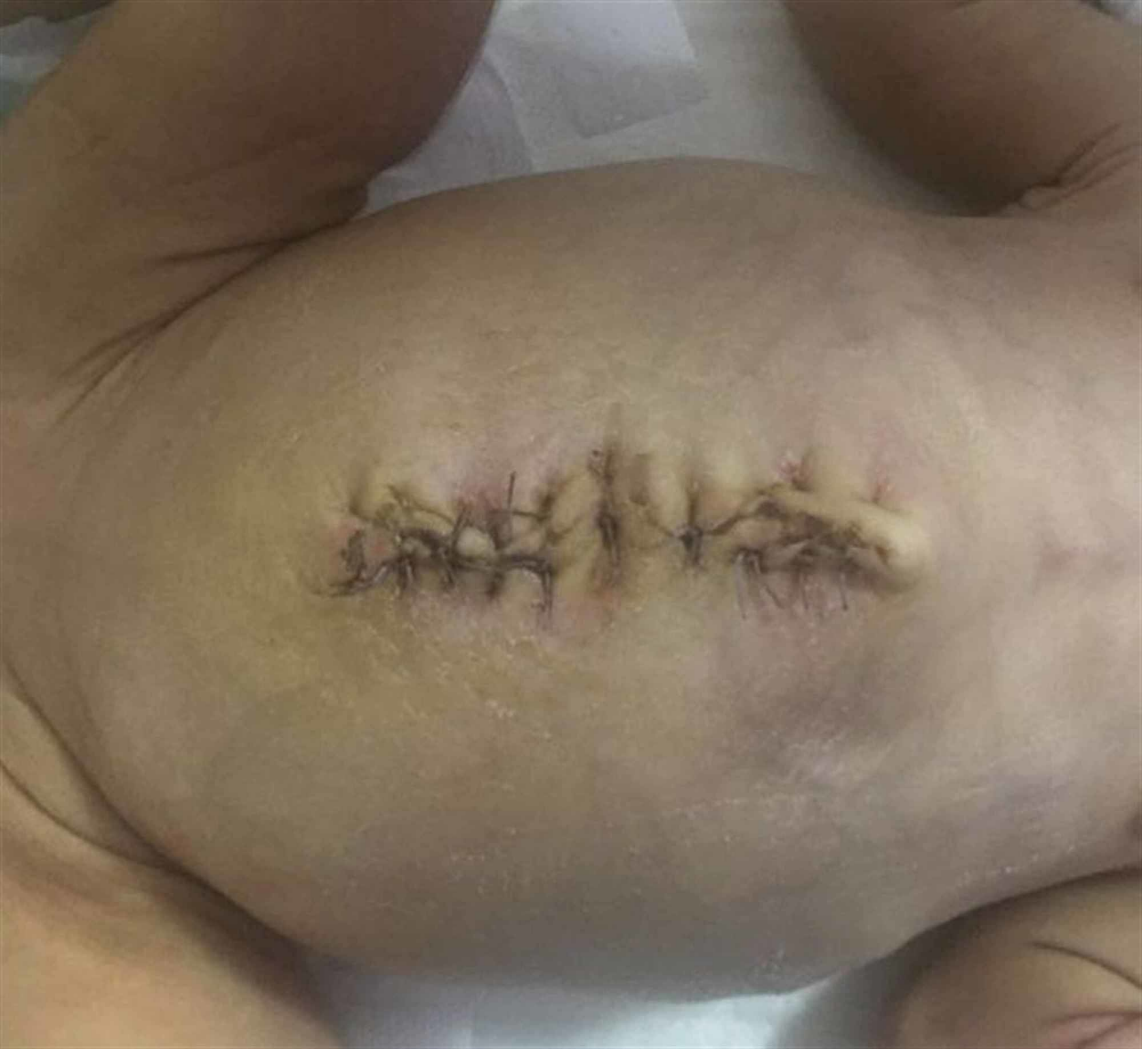
Primary omphalocele closure.

The graft was removed when the defect was thought to be closed and adequate weight gain was achieved, and the defect was closed with the primary closure method. Patients who had sufficient oral intake and had no problem in the postoperative follow-up were discharged (Figure 4).

Statistical analysis

Data obtained in the study were analysed statistically using SPSS 25.0 software (IBM Corporation, Armonk, NY, USA). The Shapiro-Wilk test was used to assess the conformity of the data to normal distribution and the variance homogeneity was evaluated with the Levene test. The Independent-Samples T test was used in conjunction with the Bootstrap results in the comparisons of quantitative data of two independent groups. The Fisher Exact test was used in conjunction with the exact results for the comparison of categorical variables with each other and the column ratios were compared with each other and expressed according to the Benjamini-Hochberg corrected p-value results. The Odds ratio was used with a 95% confidence interval to show the increased likelihood of those with a risk factor compared to those without. Quantitative variables were shown as mean ± standard deviation (SD), minimum and maximum values and categorical variables as number (n) and percentage (%) in the tables. The variables were analyzed at the 95% confidence interval and a value of p < 0.05 was accepted as statistically significant.

## Results

A total of 14 female and eight male patients were included in the present study. The mean duration of conservative monitoring was 11 days and the total hospital stay was 35 days. The mean GO defect size of the patients followed-up was 10 cm. Of the total 22 patients, nine (40.9%) had a congenital anomaly coexisting with GO, and one had Down’s syndrome.

Since seven of the patients in the present study died in the 1st week, the surgical treatment monitoring results of 15 patients were evaluated. The bodyweight of the conservative follow-up patients was determined as 3300 ± 240 grams during the defect closure. After the conservative follow-up, patients who were applied with a graft at an average of 11 days (range, 9-14 days) were hospitalized for an average of 24 days (range, 21-28 days) postoperatively. The mean duration of hospital stay of the patients was 35 days (range, 32-41 days).

The cause of death was determined as sepsis in three cases and omphalocele rupture in one. It was determined that the patients who died had a larger dimension omphalocele (p < 0.05), were not suitable for operation in the early period, had similar birth weights to those of the surviving group and died in the first week following the delivery (Tables 1, 2).

**Table 1:** Patients who underwent combined graft surgery after conservative follow-up with an elastic bandage and were applied with primary closure.

Patient No	Gender	Omphalocele Diameter	Birth Weight (g)	Body Weight During the Surgical Closure (g)	Congenital Malformation	Conservative Follow-up Time (Days)	Postoperative Follow-up Time (Days)	Mean Duration of Hospital Stay (Days)
1	Male	11	2315	3100	Absent	10	25	35
2	Male	8	2900	3600	Bilateral undescended testis	13	21	34
3	Female	13	2850	3450	PDA	14	27	41
4	Male	11	2750	3600	Absent	11	22	33
6	Male	8	2550	3000	Absent	11	23	34
8	Female	10	2880	3450	Right inguinal hernia	10	24	34
9	Female	14	2850	3500	Absent	11	25	36
11	Male	12	2300	3100	Absent	11	22	33
12	Male	9	2875	3450	Absent	11	24	35
14	Female	8	2585	3000	Absent	9	24	33
15	Male	7	2800	3600	Absent	12	28	40
18	Male	12	2740	3300	Absent	11	28	39
20	Female	9	2450	2900	Absent	10	22	32
21	Female	10	2650	3100	Down’s syndrome (Trisomy 21)	14	24	38
22	Male	11	2700	3200	Absent	11	25	36

**Table 2:** Evaluation of the patients who underwent conservative follow-up and those who died in the early postnatal period. ^f ^Fisher Exact test (Exact), ^t ^Independent samples t Test (Bootstrap), ^or ^Odds Ratio (95% Confidence interval) SD: Standard deviation, Min: Minimum, Max: Maximum

		Total		Alive		Exitus	P
				(n = 15)		(n = 7)	
		n (%)		n (%)		n (%)	
Gender							
	Female	14 (63.6)		6 (40.0)		2 (28.6)	0.999 ^f^
	Male	8 (36.4)		9 (60.0)		5 (71.4)	
Complication							
	Present	4 (18.2)		0 (0)		4 (57.1)	0.005 ^f^
	Absent	18 (81.8)		15 (100)		3 (42.9)	6 (2.1-16.8)^or^
Congenital Anomaly							
	Present	9 (40.1)		4 (26.6)		5 (71.4)	0.074 ^f^
	Absent	13 (59.9)		11 (73.4)		2 (28.6)	
		Mean ± SD (Min / Max)		Mean ± SD (Min / Max)		Mean ± SD (Min / Max)	
Defect Size (cm)		10.45 ± 2.04 (7 / 16)		10.20 ± 2.04 (7 / 14)		11.00 ± 2.83 (8 / 16)	0.030 ^t^
Birth Weight (g)		2482 ± 435 (1500 / 2950)		2679 ± 200 (2300 / 2900)		2060 ± 513 (1500 / 2950)	0.545 ^t^

## Discussion

The timing of surgical repair in patients with GO still remains controversial and there is a limited number of published studies to guide the pediatric surgeon in decision-making. Delayed closure methods have been developed to alleviate the complications of early surgical repair [14]. GO treatment is largely dependent on the size of the defect, the severity of pulmonary incompetence, and concomitant anomalies [15]. Despite advances in the fields of neonatology, anesthesia, and surgery, mortality in infants with GO reaches up to 25% and an optimal treatment method has not yet been established for these infants [6,16].

Delayed complications due to primary closure of giant omphaloceles are alarming. In a survey conducted in 2011, a review by Eijck et al. showed that the mean post-operative herniation rates were the highest in primary closure (58%) and the lowest in non-operative delayed closure (9%) [17]. However, an alternative strategy is necessary in GO cases with large tissue defects and large visceral abdominal disproportion, and in such cases, primary closure should not even be attempted.

In a review by Bauman et al., although the delayed surgical method was shown to cause prolongation of fascia closure time, increased morbidity was observed in approximately half of the patients despite administration of betadine and antisepsis in patients undergoing early surgery [18]. The primary determinant in selecting the GO closure method is the severity of visceral-abdominal disproportion. The timing of fascial defect closure depends on the growth of the infant’s abdominal cavity. However, the re-epithelization time on the omphalocele sac can be 2-3 months. If the defect is sufficiently reduced, as in small omphalocele cases, primary early fascial tissue closure can be performed (delayed closure).

Topical therapies used during the conservative follow-up in GO care for delayed closure have been greatly improved in recent years. Povidone-iodine is recommended as an effective antimicrobial alternative to silver impregnated cream. However, Betadine and Silverdin are known as the most common currently used topical drugs [19,20].

GO patients are at high risk of sac rupture, infection development, and sepsis. Therefore, topical antisepsis and regular wound care are vitally important in cases where the option of surgical closure is not possible. The combination of 5% povidone-iodine solution with topical antibiotic powder (Polymyxin, Bacitracin, and Neomycin), which has been previously used in burn patients and has an accelerating effect on wound healing, was used by Pandey et al. in 25 newborns with GO [13]. In that study, it was emphasized that antibiotic combination powder is a cost-effective treatment option that facilitates eschar formation and re-epithelization. Neomycin and Bacitracin, which are present in antibiotic spray powder combined with the betadine application, provide a barrier against microorganisms with gram-positive activity, and due to the polymyxin content, they are active against pseudomonas [21]. In addition, the powder form allows the development of scabs on the sac and facilitates the formation of a natural barrier against infections. Re-epithelization is also accelerated by the frequently changed bandage.

It is also important to ensure the position to facilitate the placement of the omphalocele sac towards the mid-abdominal line and to push the sac contents into their normal anatomical positions without increasing intra-abdominal pressure. In 2011, Sander et al. applied elastic bandage after topical treatment to patients with GO and stated that bandaging was a reliable option for re-epithelization [22]. In the present study, the abdominal elastic bandaging method was used because of its ease of application, efficacy, and reliability.

In a study by Lee et al., it was reported that most of the patients had pulmonary hypoplasia, and most had coexisting significant cardiac anomalies [23]. Ein and Langer performed delayed surgical closure with silver sulfadiazine in 20 patients with GO >10 cm in size, and reported a mortality rate of 30% [5]. In the same study, 70% of infants had pulmonary hypoplasia and 60% had a cardiac anomaly. In the present study, 13.6% of patients had a cardiac anomaly, while only one patient had pulmonary hypoplasia. The low incidence of additional congenital malformations of patients in the present study is thought to have had a positive effect on the survival of patients undergoing conservative follow-up and allowed patients to tolerate early surgery.

In our clinic, the care of hernia sacs of patients with GO is performed under sterile and routine operating room conditions. After the application of the bandage, the mothers’ breastfeeding in the early period and the follow-up of the infants in co-ordination with the nurses make it easier to evaluate the complications that may develop in hospital conditions. The incidence of omphalocele has been reported to be more frequent in the male gender, but with a slightly higher incidence of GO in females. Kumar et al. reported that female gender became more dominant as the size of omphalocele increased [24]. In the present study, GO was found to be more frequent in female gender (63.6%).

The present study has some limitations. The study was performed with a retrospective review of patient files. Therefore, it was not possible to reveal in detail the congenital malformations, infections and omphalocele sac ruptures of patients who died in the early period during the conservative follow-up with the diagnosis of GO. However, it is considered that the evaluation of hernia, complications, reoperation and survival conditions that will develop in the long-term follow-up of patients undergoing combined early surgery after conservative treatment is necessary to demonstrate the advantages of the method.

## Conclusions

In GO treatment, long-term closure is performed after non-operative follow-up. As a result of the present study, the combination of conservative waiting time and surgical closure can be considered an appropriate option in order to prevent complications that may occur during the long follow-up period. In conclusion, it is possible to reduce the length of hospital stay with primary closure in the early period by providing faster epithelialization with the combination of povidone-iodine and antibiotic (bacitracin, polymyxin, and neomycin) powder together with elastic bandage application in infants with GO.
